# Predicting Value of ALCAM as a Target Gene of microRNA-483-5p in Patients with Early Recurrence in Hepatocellular Carcinoma

**DOI:** 10.3389/fphar.2017.00973

**Published:** 2018-01-12

**Authors:** Xin-Yuan Lu, Di Chen, Xiao-Yuan Gu, Jie Ding, Ying-Jun Zhao, Qian Zhao, Ming Yao, Zhiao Chen, Xiang-Huo He, Wen-Ming Cong

**Affiliations:** ^1^Department of Pathology, Shanghai Eastern Hepatobiliary Surgery Hospital, Second Military Medical University, Shanghai, China; ^2^Key Laboratory of Signaling Regulation and Targeting Therapy of Liver Cancer of Ministry of Education, Second Military Medical University, Shanghai, China; ^3^Shanghai Key Laboratory of Hepatobiliary Tumor Biology, Eastern Hepatobiliary Surgery Hospital, Shanghai, China; ^4^Fudan University Shanghai Cancer Center and Institutes of Biomedical Sciences, Shanghai Medical College, Fudan University, Shanghai, China; ^5^Department of Oncology, Shibei Hospital of Shanghai, Shanghai, China; ^6^State Key Laboratory of Oncogenes and Related Genes, Shanghai Cancer Institute, Renji Hospital, Shanghai Jiao Tong University School of Medicine, Shanghai, China

**Keywords:** ALCAM, miR-483-5p, hepatocellular carcinoma, early recurrence, invasion and metastasis, EMT

## Abstract

The long-term survival rate of hepatocellular carcinoma (HCC) is poor. One of the reasons for the poor rate of survival is the high rate of recurrence caused by intrahepatic metastas is that adversely affects long-term outcome. Many studies have indicated that microRNAs play an important role in HCC, but there has been no research of clonal origins on recurrent HCC (RHCC) by analzing microRNAs. In the present study, we found that miR-483-5p was significantly upregulated in RHCC tissues of short-term recurrence (≤ 2 years) by miRNA microarray screening, and can significantly promote migration and invasion of HCC cells *in vitro* and increase intrahepatic metastasis in nude mice *in vivo*. Furthermore, we demonstrated that activated leukocyte cell adhesion molecule (ALCAM), which significantly suppressed migration and invasion of HCC cells, was a direct target of miR-483-5p, and the re-introduction of ALCAM expression could antagonize the promoting effects of miR-483-5p on the capacity of HCC cells for migration and invasion. In addition, expression level of ALCAM was negatively correlated with microvascular invasion and tumor size recognized as prognostic factors. The cases which were negative for ALCAM expression had shorter time to recurrence than positive cases, and univariate and multivariate survival analyses showed that ALCAM was an independent risk factor of HCC recurrence. qRT-PCR and Western blotting showed that the expression of EMT related genes (MMP-2, MMP-9, E-caherin and vimentin) significantly changed as a result of interfering or overexpression of ALCAM, and ALCAM was significantly associated with EMT in HCC. These results suggest that the miR-483-5p/ALCAM axis is an important regulator in invasion and metastasis and biomarker for recurrence risk assessment of HCC.

## Introduction

Hepatocellular carcinoma (HCC) is the fifth most frequently diagnosed cancer, and the third leading cause of cancer death in the world. An estimated 782,500 new liver cancer cases and 745,500 deaths occurred worldwide during 2012, with China alone accounting for approximately 50% of the total number of cases and deaths (Torre et al., 2015). Surgical resection remains the first choice of treatment of HCC; however, the long-term survival rate is poor. One of the reasons for the poor rate of survival is the high rate of recurrence caused by intrahepatic metastasis that adversely affects long-term outcome. A greater understanding of the molecular mechanisms under lying recurrence and intrahepatic metastasis of HCC may have a significant effect on improving prognosis and systematic treatment of this disease.

MicroRNAs (miRNAs) are small, noncoding RNAs of 21 to 25 nucleotides in length that direct post-transcriptional regulation through specific recognition of short sequences of target messenger RNAs (mRNAs), often in the 3′untranslated region (3′-UTR), causing either target mRNA degradation or inhibition of translation through assembling the RNA-induced silencing complex. Accumulating evidence suggests that the levels of miRNAs are deregulated and can play an important role in evolution and progression of HCC (Yang et al., 2016), especially by affecting invasion and metastasis of tumor cells. In HCC, Let-7g and miR-122 inhibit cell migration and intrahepatic metastasis (Tsai et al., 2009; Ji et al., 2010), whereas miR-143, miR-16, miR-30a, let-7e and miR-204 can significantly promote HCC metastasis (Zhang et al., 2009; Zeng et al., 2012). However, the variation of miRNAs in recurrent HCC (RHCC) caused by intrahepatic metastasis is rarely reported, and the underlying molecular mechanisms for intrahepatic metastasis remain unclear.

We selected postoperative RHCC cases, which were divided into two groups: short-term recurrence (≤ 2 years) and long-term recurrence (>2 years). The miRNA expression profiles were determined with miRNA array, and five miRNAs were found to be different between the two groups. miR-483-5p was the most significantly different miRNAs. The function and mechanism of miR-483-5p in RHCC are not clear; hence, we performed gain- and loss-of-function studies to determine the biological roles of miR-483-5p in this study, and we integrated bioinformatics predictions, expression datasets, and luciferase reporter assay results to reveal its underlying molecular mechanism in HCC. We found that miR-483-5p promotes tumor invasion in HCC, andactivated leukocyte cell adhesion molecule (ALCAM) is characterized as a direct and functional target of miR-483-5p in HCC cells. These findings indicate that the miR-483-5p/ALCAM axis is an important regulator in intrahepatic metastasis of HCC and can serve as prognostic markers and basis of individualized treatment.

## Materials and methods

### Human specimens

All RHCC cases used in this study were obtained during liver resections performed in the Eastern Hepatobiliary Surgery Hospital (Shanghai, China) from 2000 to 2012. microsatellite LOH was detected in the primary-recurrence tissue samples to determine the clonal origin of RHCC (Wang et al., 2013), and the monoclonal recurrent cases were divided into two groups according to interval time between primary and recurrence: short-term recurrence (less than 2 years [G1]) and long-term recurrence (more than 2 years [G2]). These samples were obtained with informed consent according to the guidelines set forth by the Eastern Hepatobiliary Surgery Hospital Research Ethics Committee.

### miRNA extraction and array screening

Total miRNA was extracted from formalin-fixed paraffin-embedded (FFPE) tissues using miRNeasy FFPE Kit (QIAGEN, Germany). The miRNA expression profiles were determined with Agilent miRNA Microarray (Agilent Human miRNA V16.0), and eight cases of RHCC were choose for miRNAs array testing (four cases in each group). For miRNA detection, mature miRNA was reverse-transcribed and quantified with TaqMan® RT primers and probe, and normalized to U6 small nuclear RNA expression, using predesigned TaqMan assays (Applied Biosystems, Foster City, California, USA).

### Cell culture

Cell lines used in this study were SK-Hep1, SMMC-7721, Huh7, and HEK 293T, and all cells were cultured in Dulbecco modified Eagle medium (DMEM; Gibco, NewYork, USA); The media were supplemented with 10% fetal bovine serum (Gibco), 100 IU/ml penicillin G, and 100 μg/mL streptomycin sulfate (Sigma-Aldrich, St. Louis, Missouri, USA) in a humidified 37°C incubator with 5% CO_2_.

### RNA extraction and real-time quantitative polymerase chain reaction analysis

Total RNA in cells was extracted by applying the TRIzol® reagent (Invitrogen, Carlsbad, California, USA). Reverse-transcribed complementary DNA was synthesized with the Prime-Script® RT Reagent Kit (TaKaRa, Tokyo, Japan). Quantitative polymerase chain reaction analyses were performed with LightCycler®480 SYBR Green I Master (Roche, Welwyn Garden, Swiss). To detect mature miRNA, RNA was reverse-transcribed and quantified with TaqMan® RT primers and probe, and normalized to U6 small nuclear RNA expression, using predesigned TaqMan assays (Applied Biosystems).

### Oligonucleotide transfection

miR-483-5p mimics and ALCAM small interfering RNA (siRNA) duplexes were designed and synthesized by RiboBio (Guangzhou, China), and miR-483-5p inhibitors were designed and synthesized by GenePharma (Shanghai, China). Cells were transfected in individual wells of six-well plates with a mimic, an inhibitor, or an siRNA pool (three siRNAs were mixed in an equimolar ratio) targeting miR-483-5p by using Lipofectamine® 2,000 at a final concentration of 50 nM. At 48 h posttransfection, the cells were harvested for the assays described later.

### Lentivirus packaging and infection

Lentivirus particles were harvested 48 h after pWPXL-483-5p or pWPXL- ALCAM cotransfection with the packaging plasmid psPAX2 and the vesicular stomatitis virus G VSV-G envelope plasmid pMD2.G (psPAX2 and pMD2.G were gifts from Dr. Didier Trono) into HEK293T cells using Lipofectamine^®;^ 2000. Cells were infected with the resultant recombinant lentivirus in the presence of 6 μg/mL polybrene (Sigma-Aldrich).

### *In vitro* cell proliferation, migration, and invasion assays

One thousand cells were placed in a fresh 96-well plate in triplicate and maintained in DMEM containing 10% fetal bovine serum for 5 days, and cell proliferation was measured with the Cell Counting Kit-8 (Dojindo, Kumamoto, Japan) following the manufacturer's instructions.

For transwell migration assays, 5 × 10^4^ cells were plated in the top chamber of each insert (BD Biosciences) with a non-coated membrane. For invasion assays, 1 × 10^5^ cells were added to the upper chamber with 150 μg Matrigel (BD Biosciences). For both assay types, 800 μL of medium supplemented with 10% fetal bovine serum was injected into the lower chambers. After harvest, the inserts were fixed and stained in a dye solution containing 0.1% crystal violet and 20% methanol. Imaging of cells adhering to the lower membrane of the inserts was performed with an IX71 inverted microscope (Olympus, Tokyo, Japan), and five randomly selected fields were quantified.

### *In vivo* liver orthotopic transplantation

For *in vivo* metastasis assays, 2 × 10^6^ SMMC-7721 cells infected with miR-483-5p or mock vector, respectively, were suspended in 40 μL serum-free DMEM/Matrigel (1:1) for each mouse. Through an 8-mm transverse incision in the upper abdomen under anesthesia, each nude mouse (12 female BALB/c-nu/nu in each group) was orthotopically inoculated in the left hepatic lobe with a microsyringe. After 10 weeks, the mice were sacrificed, and their livers and lungs were dissected, fixed with phosphate-buffered neutral formalin, and prepared for standard histological examination. The mice were manipulated and housed according to protocols approved by the Shanghai Medical Experimental Animal Care Commission.

### Luciferase assays

HEK 293T cells were cultured in 96-well plates and cotransfected with 20 ng of the psiCHECK-2-ALCAM-3′-UTR vector and either 5 pmol of the miR-483-5p mimics or the control mimics. After 48 h of incubation, firefly and Renilla luciferase activities of the cell lysates were measured using the Dual-Luciferase Reporter Assay System (Promega).

### Tissue microarray, immunohistochemistry, and scoring

Anti-ALCAM was purchased from Abcam (Cambridge, UK). The tissue microarray was constructed as described previously (Zhu et al., 2008). Core samples were obtained from representative regions of each tumor based on hematoxylin and eosin staining. Duplicate 1.5-mm cores were taken from different areas of the same tissue block for each case (intratumoral tissue and peritumoral tissue). Serial sections (4 μm thick) were placed on slides coated with 3-aminopropyltriethoxysilane. The immunohistochemistry analysis was carried out as described previously (Lu et al., 2011). The primary monoclonal antibody used was rabbit anti-human (1:100). The positive staining of ALCAM was located at the cellular membrane and the immunostaining intensities were scored semiquantitatively as follows: 0 = negative; 1 = positive. All samples were anonymously and independently scored by two investigators (XY-Lu and WM-Cong). In case of disagreement, the slides were reexamined and consensus reached by the observers. The intensity of immunostaining was scored on the basis of the percentage of positive tumor cells: 0 (–) (0–5%), 1 (+) (6–25%), 2 (++) (26–50%), and 3 (+++) (>51%) for ALCAM.

### Statistical analysis

Results are presented as mean ± standard error of the mean from at least three independent experiments. Unless otherwise stated, differences between two groups or more than two groups were determined using Student *t*-test or one way analysis of variance, respectively, followed by Dunnett's multiple-comparison test. The cumulative recurrence and survival rates were determined using the Kaplan-Meier method (log-rank test). The Cox multivariate proportional hazards regression model was used to determine the independent factors that influence survival and recurrence based on the investigated variables. Values of *p* < 0.05 were considered statistically significant. Statistical analyses were performed using GraphPad Prism, version 6.00 for Windows (GraphPad Software, LaJolla, California, USA) and IBM SPSS Statistics 18.0 (Armonk, NY, USA).

## Results

### miR-483-5p is upregulated in RHCC patients with short-term recurrence

**Figure 1:**
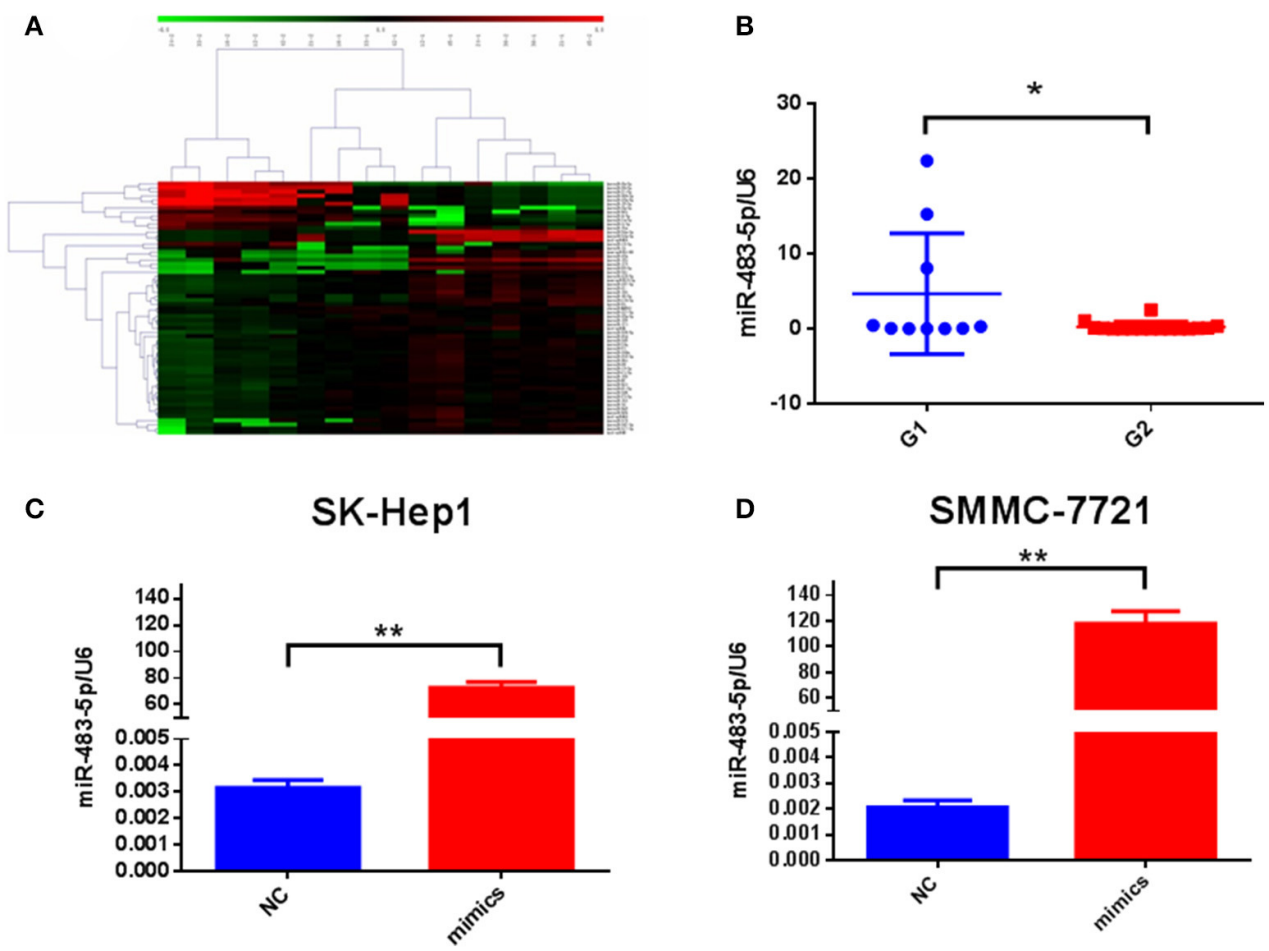
Expression of miR-483-5p in RHCC tissues and cells. **(A)** miRNAs array screened miRNA expression profiles. **(B)** Differential expression of miR-483-5p between two groups. **(C,D)** Transfection efficiency of miR-483-5p mimics in SK-Hep1/SMMC-7721 cells. ^*^p < 0.05; ^**^p < 0.01.

To find differential expressed miRNAs and potential predictive markers in HCC recurrence, we first determined the expression of miRNAs with miRNA array between short-term recurrence (G1, ≤ 2 years) and long-term recurrence group (G2, > 2 years), and five upregulated miRNAs (hsa-miR-133a, hsa-miR-4251, hsa-miR-4279, hsa-miR-483-5p, hsa-miR-642b-3p) were identified (Figure 1A). To validate the miRNA array data, real-time quantitative polymerase chain reaction analysis was performed using RHCC tissue. Results showed that miR-483-5p was the most significantly different miRNA between the two groups, and it was selected for further studies (Figure 1B). These results suggested that upregulation of miR-483-5p is related to the short-term recurrence of HCC, and may play an important role in the metastasis and recurrence process.

### miR-483-5p promotes HCC cell invasion and metastasis *in vitro* and *in vivo*

To better understand the biological functions of miR-483-5p expression on HCC recurrence, we synthesized miR-483-5p mimics and inhibitor and constructed a lentivirus vector expressing miR-483-5p for a functional experiment.

**Figure 2:**
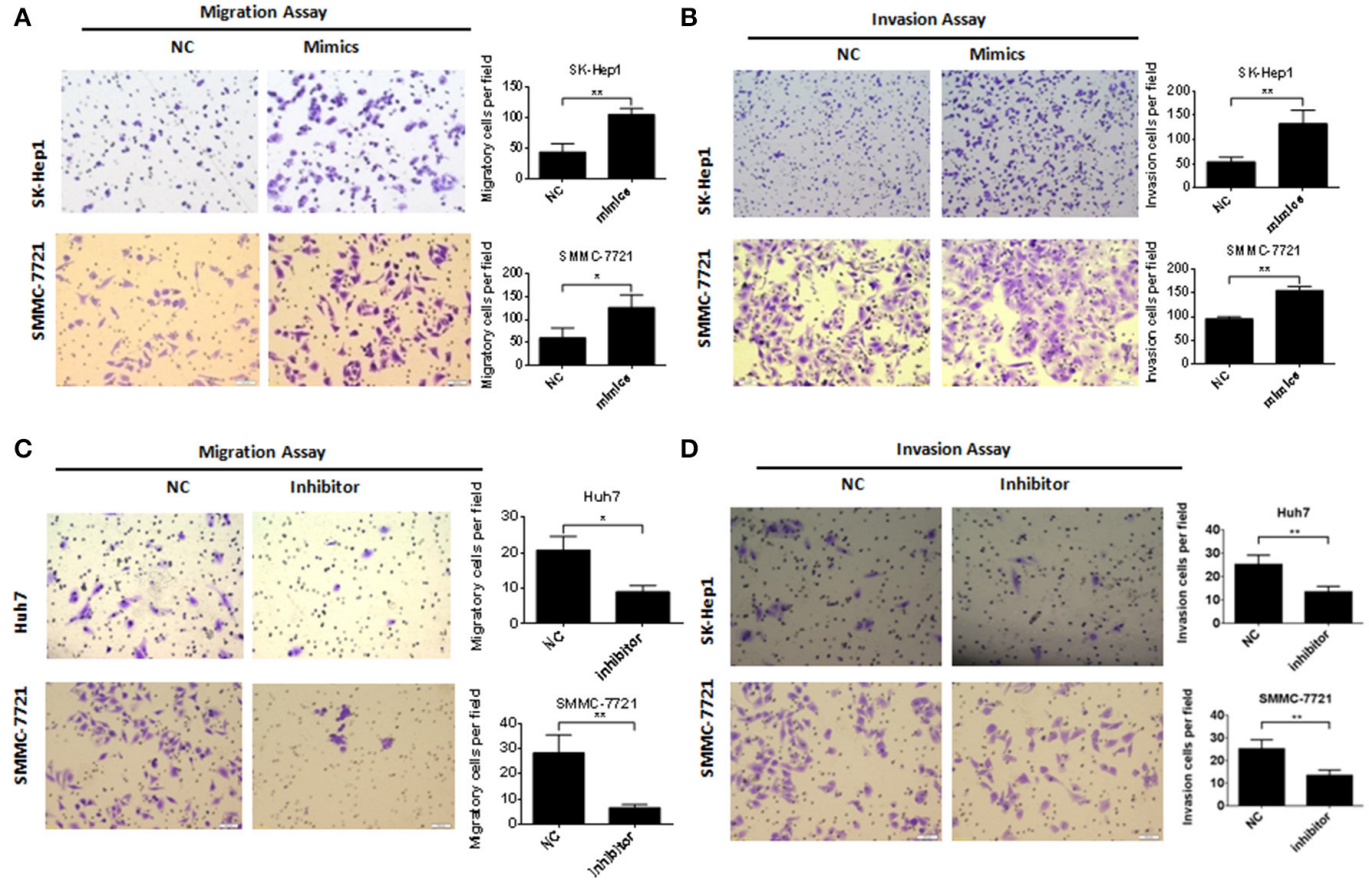
miR-483-5p promoted HCC cells migration and invasion. **(A)** miR-483-5p mimics promoted HCC cells migration. **(B)** miR-483-5p mimics promoted HCC cells invasion. **(C)** miR-483-5p inhibitor suppressed HCC cells migration. **(D)** miR-483-5p inhibitor suppressed HCC cells invasion. ^*^p < 0.05; ^**^p < 0.01 ×40.

We transiently transfected miR-483-5p mimic into SK-Hep1 and SMMC-7721 (Figures 1C,D), and transfected miR-483-5p inhibitor into SMMC-7721 and Huh7. During cell proliferation assay, ectopic expression of miR-483-5p had no obvious effects on HCC cell proliferation (Figures S1A,B). Because the expression of miR-483-5p was highly associated with the recurrence and metastasis of HCC in the preliminary miRNA microarray results, we wondered whether miR-483-5p could play an important role in HCC cell invasion and metastasis. Transwell assays without Matrigel results showed that miR-483-5p mimics dramatically promoted the migration of SK-Hep1 and SMMC-7721 cells when compared with the indicated controls (Figure 2A). Transwell assays with Matrigel demonstrated that miR-483-5p mimics dramatically promoted the invasive capacities of these two cell lines when compared with the vector control groups (Figure 2B). Furthermore, the migration and invasion of Huh7 and SMMC-7721 cells decreased when endogenous miR-483-5p was silenced with an inhibitor (Figures 2C,D). Our results illustrated that miR-483-5p can significantly enhance HCC cell migration and invasion *in vitro*.

To further reveal the role of miR-483-5p in tumor invasion and metastasis *in vivo*, we used a lentivirus system to establish a stable SMMC-7721 cell line with miR-483-5p overexpression, and SMMC-7721 cells with stable GFP overexpression served as a control. These cell lines were designated as Lenti-miR-483-5pand Lenti-GFP, respectively. Next, Lenti-miR-483-5p and Lenti-GFP were transplanted into the livers of nude mice. The HCC cell line SMMC-7721 has been employed in *in vivo* metastasis assays in nude mice, including orthotopical liver implantation for intrahepatic metastasis and distant metastasis, because it has relatively strong *in vitro* invasive properties (Liu, 2008).

**Figure 3:**
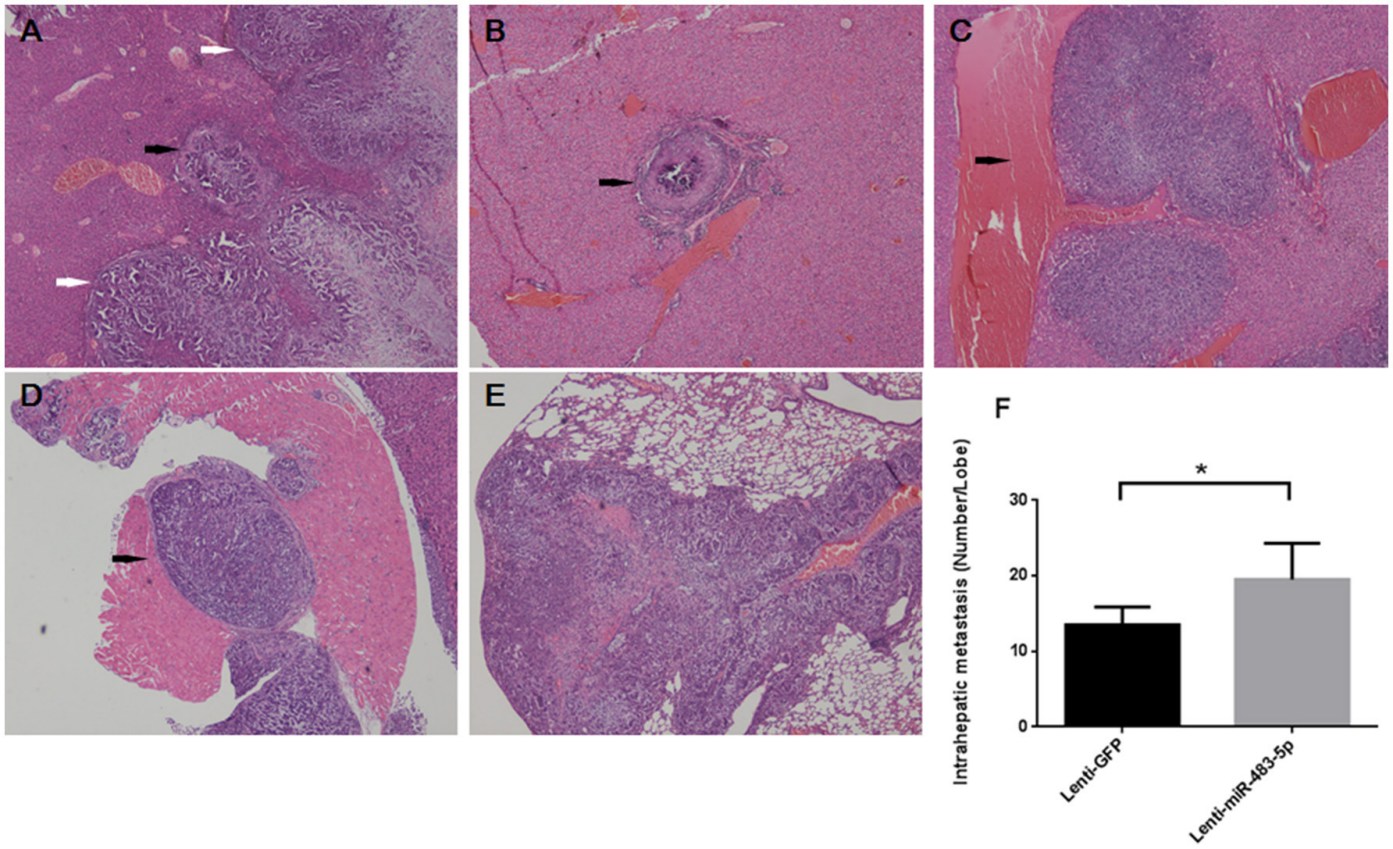
miR-483-5p promotes HCC cell invasion and metastasis *in vivo*, **(A–F)** showed intrahepatic metastasis and distant metastasis (lung) in Lenti-miR-483-5p mice. **(A)** Orthotopic primary liver tumor (white arrows) and intrahepatic metastatic nodules close beside tumor (black arrow). **(B)** Distant microvascular invasion in another liver lobe (black arrow). **(C)** Intrahepatic metastatic nodules close beside larger vein branch (black arrow). **(D)** Metastatic nodules invasion smooth muscular tissue near liver (black arrow). **(E)** Distant metastasis in the lung. **(F)** The numbers of metastatic nodules in the livers of each mouse are counted; the statistical significance is labeled using the 2 test. ^*^p < 0.05, HE stained ×40.

Orthotopic liver implantation results showed that the number of metastatic nodules in the liver and lung were dramatically increased in the Lenti-miR-483-5pgroup compared with that in the vector control group after 10 weeks. Intrahepatic metastasis was observed in eight mice of the Lenti-miR-483-5p group and four mice of the vector control group, and pulmonary metastasis occurred in three Lenti-miR-483-5p mice compared with none in the vector control group (30% vs. 0, *p* = 0.030). Interestingly, additional invasive expression was observed in Lenti-miR-483-5pmice, along with common intrahepatic metastatic nodules (Figure 3A), such as intrahepatic multiple metastatic nodules and distant primary lesion metastasis (Figure 3B), vascular invasion close beside larger vein branch (Figure 3C), metastatic nodules invasion smooth muscular tissue (Figure 3D), and distant metastasis in the lung (Figure 3E). The number of metastatic nodules in the livers of Lenti-miR-483-5p group is significantly higher than that of vector control (Figure 3F). These results suggest that miR-483-5p is a positive invasive and metastatic regulator for HCC.

### miR-483-5p downregulates ALCAM expression by directly targeting its 3′-UTR

**Figure 4:**
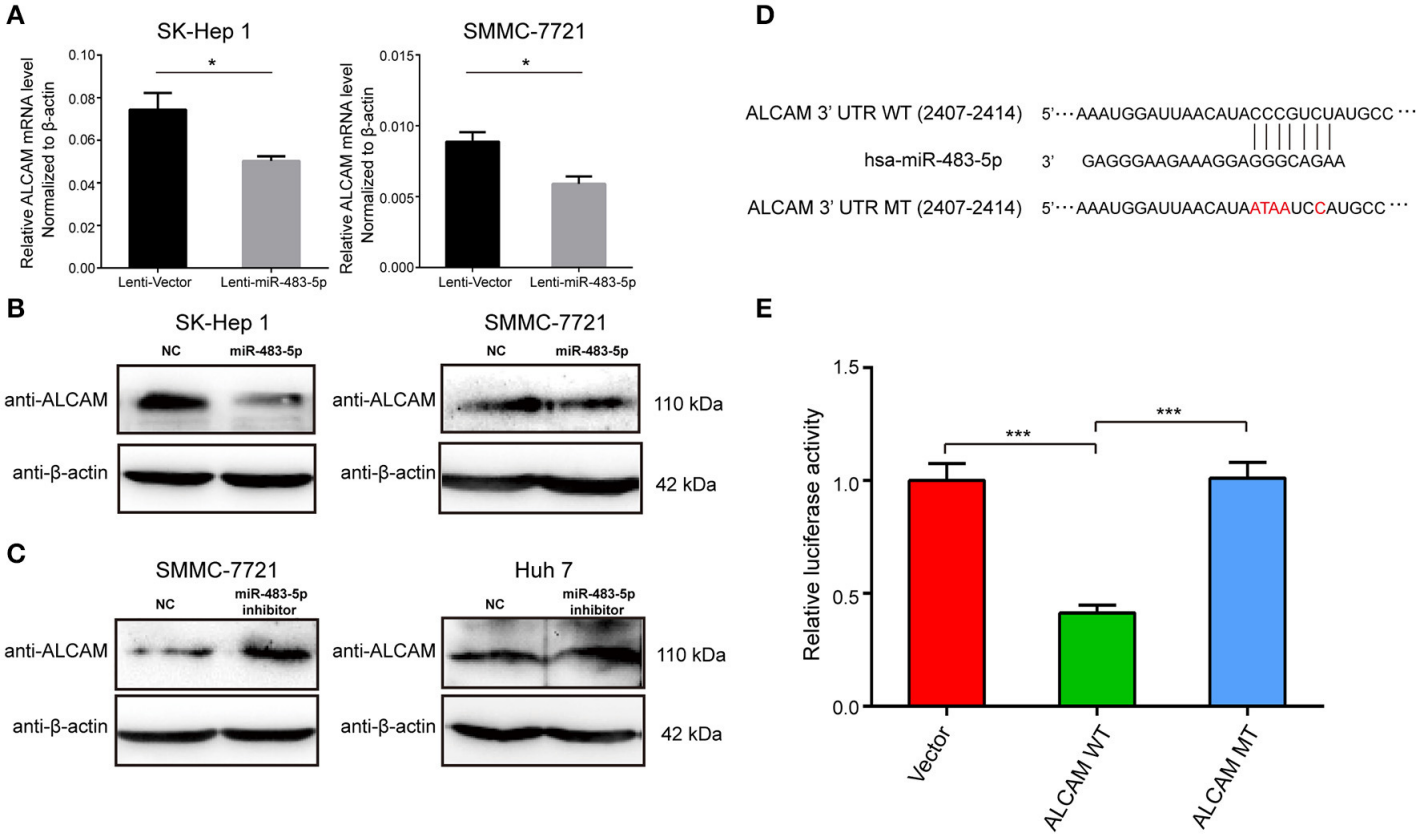
miR-483-5p post-transcriptionally downregulates ALCAM expression by directly targeting its 3′-UTR. **(A)** ALCAM mRNA levels were repressed by miR-483-5p in HCC cells. **(B)** ALCAM protein levels were repressed by miR-483-5p mimics in HCC cells. **(C)** ALCAM protein levels were up-regulated in HCC cells with miR-483-5p inhibitor. **(D)** Diagram of putative miR-483-5p binding sites in the 3′-UTR of ALCAM. The mutant sequences ALCAM 3′-UTR used in the luciferase reporter constructs are indicated in red. **(E)** Relative activities of luciferase reporters containing ALCAM 3′-UTR variants co-transfected with miR-483-5p or negative control mimics in HEK 293T cells. ^*^p < 0.05; ^***^p < 0.001.

To elucidate the underlying molecular mechanism through which miR-483-5p initiates HCC cell invasion and metastasis, we used the public prediction algorithm TargetScan (http://www.targetscan.org) to explore potential targets for miR-483-5p. A total of 55 candidate mRNA targets were found (Table S1). When candidate genes were detected in Lenti-miR-483-5p cells with qPCR, we found that of 55 predicted candidate genes in two cells, qPCR (Figure 4A) and western blot (Figures 4B,C) analysis demonstrated that the expression of miR-483-5p was negatively correlated with ALCAM at both the mRNA and protein levels. To determine whether ALCAM is regulated by miR-483-5pthrough direct binding to its 3′-UTR, a series of 3′-UTR fragments, including full-length 3′-UTR, binding site (wild-type and mutant) (Figure 4D),were constructed and inserted into the region immediately downstream of the luciferase reporter gene.

In 3′-UTR luciferase reporter assays, we confirmed that miR-483-5p could directly bind to sites (nucleotides 2407-2414) in the 3′- UTR of ALCAM. Mutations in miR-483-5p recognition site rendered the constructs unresponsive to miR-483-5p induction, indicating the binding between miR-483-5p and the site in ALCAM 3′-UTR region (Figures 4D,E).

### ALCAM inhibits HCC cell migration and invasion

**Figure 5:**
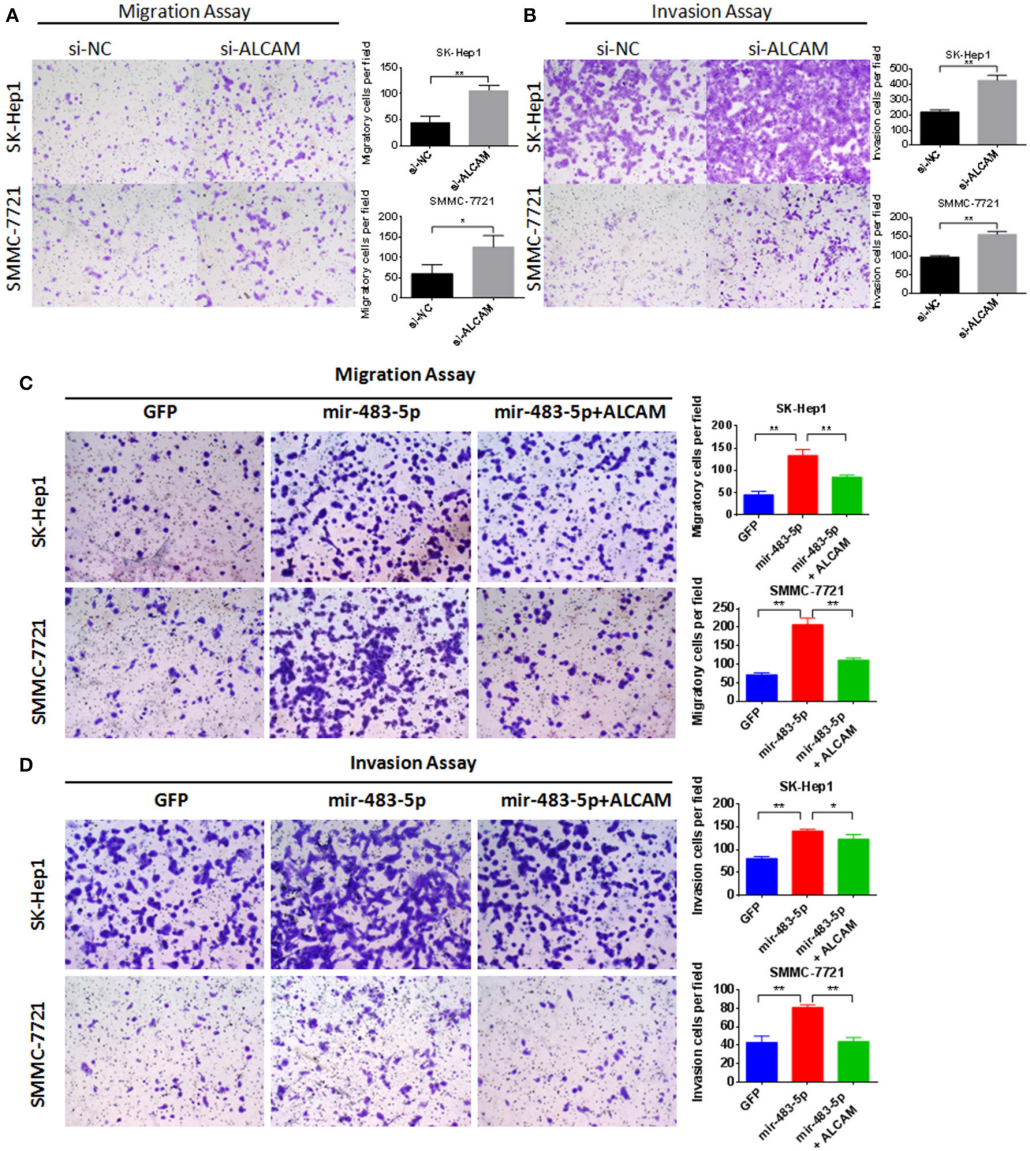
Knockdown of ALCAM can promote HCC cell migration and invasion. Restoration of ALCAM reverses miR-483-5p-mediated HCC cell migration and invasion. **(A)** Transwell migration assay of SK-Hep1 and SMMC-7721 showed transfection of ALCAM siRNAs promoted cell migration. **(B)** Transwell invasion assay showed transfection of ALCAM siRNAs promoted cell invasion. **(C)** The migration of HCC cells induced by miR-483-5p was restored via ALCAM re-introduction. **(D)** The invasion of HCC cells induced by miR-483-5p was restored via ALCAM re-introduction. ^*^p < 0.05; ^**^p < 0.01 ×40.

To better understand the potential role of ALCAM in miR-483-5p-mediated tumor invasion and metastasis, we performed gain-of-function and loss-of-function analyses. SK-Hep1 and SMMC-7721cells were transfected with ALCAM siRNAs or a negative control siRNA (Table S2). Results from transwell assays showed that knockdown of ALCAM dramatically promoted cell migration (Figure 5A) and invasion (Figure 5B) in both SK-Hep1 and SMMC-7721 cells. However, transfection with si-ALCAM did not affect cell proliferation in both cells. During cell proliferation assay, knockdown of ALCAM had no obvious effects on HCC cell proliferation (Figures S1C,D). These results were consistent with the promotion effects on HCC cell observed with miR-483-5p mimics or stable overexpression.

### Restoration of ALCAM inhibits miR-483-5p-mediated HCC cell migration and invasion

Because si-ALCAM can promote HCC cell migration and invasion, and miR-483-5p can post-transcriptionally regulate the expression of ALCAM by directly binding to its 3′-UTR, we hypothesized that the downregulation of ALCAM directly mediates miR-483-5p-initiated HCC invasion and metastasis. To further address this critical issue, we constructed a lentivirus plasmid containing the ALCAM complementary DNA sequence without its 3′-UTR, which enabled constitutive ALCAM expression without the potential miR-483-5p binding sites. Reintroduction of ALCAM significantly reversed miR-483-5p-induced promotion of migration and invasion (Figures 5C,D). In summary, these data demonstrate that ALCAM is a direct and functional target for miR-483-5p.

### Clinical significance and prognostic analysis of ALCAM

**Figure 6:**
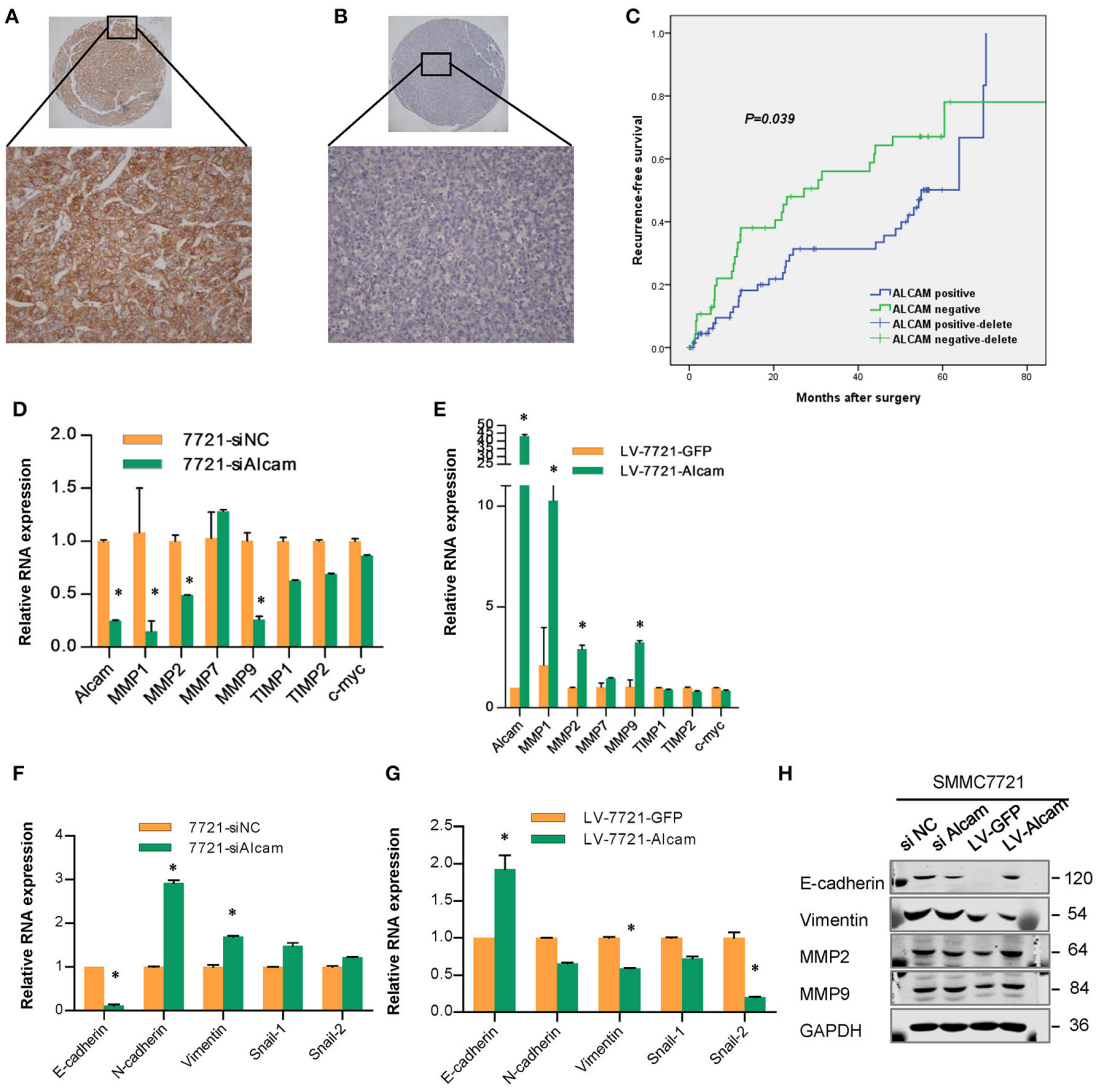
Immunohistochemistry staining and microarray analysis of ALCAM in HCC patients and relationship of ALCAM and EMT related genes. **(A)** The positive staining of ALCAM was mainly located at the cellular membrane, compared with negative staining in HCC **(B)**; **(C)** Kaplan-Meier analysis of the correlation between ALCAM expression and the recurrence-free of patients with HCC. Log-rank tests were used to determine statistical significance. The expression of ALCAM was associated with the recurrence-free survival of patients with HCC, ALCAM negative cases had worse TTR than positive cases (log-rank test, *p* < 0.05). **(D–G)** Relative mRNA levels as measured by qPCR of the EMT related genes in SMMC-7721 cells transfected with si-NC or si-ALCAM **(D,F)** and infected with control and LV-ALCAM **(E,G)**. **(H)** Western blot showed that the expression of ALCAM and the EMT markers in SMMC-7721 cells with ALCAM siRNAs or stably transfected with Lenti-ALCAM. ^*^p < 0.05.

To determine the clinicopathologic significance of ALCAM in HCC, we obtained FFPE HCC tissues from 129 patients with HCC who had undergone curative resection and tissue microarray was constructed for further analyses. The positive staining of ALCAM was located at the cellular membrane or cytoplasm in immunohistochemistry stains, analysis results showed that 59.3% cases were positive on the cellular membrane, and 12.5% cases were positive in cytoplasm. ALCAM level was determined by immunohistochemistry on tissue microarray, and the patients were divided into two groups (negative and positive) according to the membrane expression of ALCAM in the HCC tissue (Figures 6A,B). Clinical pathological data was statistically analyzed between the two groups. We found that the expression level of ALCAM was negatively correlated with microvascular invasion and tumor size. However, ALCAM overexpression was correlated with liver cirrhosis (Table 1).

**Table 1:** Relationship of ALCAM expression level with the clinicopathological features in HCC.

**Characteristics**	**Positive**	**Negative**	***p***
**AGE, YEARS**			
≤50	36	21	0.726
>50	36	24	
**GENDER**			
Male	64	36	0.246
Female	4	5	
**HBsAg**			
–	1	0	0.427
+	71	45	
**HBV-DNA**			
–	15	7	0.541
+	47	30	
**AFP**			
–	27	11	0.129
+	44	34	
**LIVER CIRRHOSIS**			
–	51	40	0.029^*^
+	20	5	
**TUMOR SIZE (cm)**			
≤3	13	17	0.042^*^
>3	58	32	
**MICROVASCULAR INVASION**			
–	60	28	0.016^*^
+	12	16	
**CAPSULAR INVASION**			
–	31	23	0.321
+	36	18	
**TUMOR DIFFERENTIATION**			
I–II	12	6	0.627
III–IV	60	39	

Statistical analyses were done by the Chi-square (χ2) test. (

* *p < 0.05)*.

*AFP, Alpha fetoprotein*.

In addition, we evaluated the clinical relevance of ALCAM expression to prognosis in the cohort. At the time of last follow-up, of the 129 patients studied, 66 had tumor recurrence and 52 died. Kaplan–Meier analysis showed that the 1,3, and 5-year survival rates for ALCAM-positive cases were 75.8%, 60.1%, and 54.1%, respectively, and the survival rates for the ALCAM-negative cases were 80%, 56.3%, and 50.2%, respectively; median overall survival (OS) time was 37.8 months for patients who were positive for ALCAM and for ALCAM-negative cases (*p* > 0.05, log rank test). The 1,3, and 5-year recurrence rates of ALCAM-positive cases were 16.4, 31.3, and 50.1%, respectively, and the recurrence rates for the negative cases were 33.5, 56.1, and 67%, respectively. The median time to recurrence (TTR) was 54.9 months for patients who were positive for ALCAM and 27.2 months for ALCAM-negative cases (*p* < 0.05, log rank test, Figure 6C). The data indicate recurrence-free survival was poorer in patients with ALCAM-negative expression than in those with ALCAM-positive expression.

To test whether the expression levels of ALCAM were independent of other predictive variables, we applied univariate and multivariate analyses using a Cox multivariate proportional hazard regression model with ALCAM expression and clinicopathologic factors (such as age, sex, hepatitis B virus, tumor size, vascular invasion, and tumor differentiation) as covariates. Univariate analysis showed that hepatitis B e-antigen, hepatitis B e-antibody (HBeAb), and the expression of ALCAM were the factors that were consistently significant for TTR, and HBeAb, alpha-fetoprotein (AFP), albumin, and vascular invasion were consistently significant for OS (Table S3). A multivariate statistical analysis revealed that ALCAM-negative cases harbored a 1.876-fold higher risk of cancer recurrence (*p* = 0.022, 95% confidence interval 1.096–3.211) than ALCAM- positive cases, and ALCAM was an independent prognostic factor for TTR (Table 2). HBeAb, vascular invasion, and AFP were independent prognostic factors for OS.

**Table 2:** Multivariate analysis of factors influencing overall survival (OS) and time to recurrence (TTR).

**Independent factors**	***p*-value**	**HR**	**95% CI for Exp(B)**	
			**Lower**	**Upper**
**OS**				
HBeAb (Pos vs. Neg)	0.007^**^	0.430	0.201	0.625
AFP (Pos vs. Neg)	0.102	1.866	1.083	2.915
Serum albumin (low vs. nor)	0.001^**^	0.345	0.143	0.813
Vascular invasion (yes vs. no)	0.033^*^	2.009	1.259	3.321
**TTR**				
ALCAM (Neg vs. Pos)	0.022^*^	1.876	1.096	3.211
HBeAg (Pos vs. Neg)	0.955	0.971	0.353	2.674
HBeAb (Pos vs. Neg)	0.084	0.406	0.146	1.130

*OS, Overall survival; TTR, Time to recurrence*.

* *p < 0.05*,

** *p < 0.01*.

The epithelial-mesenchymal transition (EMT) is a key step in cancer recurrence and metastasis by which epithelial cells lose their cell polarity and cell-cell adhesion, and gain migratory and invasive properties to become mesenchymal cells. The invasion and metastasis of HCC are closely related to tumor cell EMT, study has shown that the decrease in ALCAM expression was accompanied by a significant upregulation of MMP-2 expression in breast cancer (Jezierska and Motyl, 2009). When the expression of EMT molecules were studied with the HCC cell lines by qRT-PCR and Western blotting, we observed that the expression of EMT related genes (MMP-2, MMP-9, E-caherin and vimentin) significantly changed as a result of interfering or overexpression of ALCAM (Figures 6D–H). This result further confirms that ALCAM is significantly associated with EMT in HCC cells, miR-483-5p-ALCAM can affect the invasion and metastasis of HCC by regulating the EMT process of HCC cells.

## Discussion

HCC is a common malignant tumor with a high mortality rate. Currently, surgical resection is still the most effective treatment for HCC, but the prognosis for HCC patients is unsatisfactory because of biologic characteristics such as strong invasiveness, recurrence, and metastasis. More than 90% of deaths in cancer patients are related to recurrence and metastasis; the 5-year recurrence rate is as high as 60% to approximately 90% after operation, which has restricted the long-term efficacy of surgical resection (Cong, 2016). Recurrence also occurs in the short term in clinical studies, which seriously affects the disease-free survival time and the treatment effect. The results of molecular pathology studies on the clonal origin of the RHCC showed that there were two models of origin of RHCC: intrahepatic metastasis and multicentric occurrence (Wang et al., 2013). Treatment method should be selected according to different recurrence types; theoretically, the curative effect of reoperation to multicentric occurrence cases is similar to the primary tumor recurrence, whereas interventional treatment is more suitable for intrahepatic metastasis cases. The clonal origin research has limited value for the assessment of recurrence risk. To determine the recurrence risk prediction markers, the ability to understand, predict, and reduce the recurrence of HCC is necessary.

There are differences in the expression level of miR-483-5p in HCC tissue with different recurrence intervals, the *in vivo* and *in vitro* experiments confirmed that the high expression of miR-483-5p could promote the migration and invasion of HCC cells, and this effect is directly targeting ALCAM.

Currently, research on miR-483-5p of HCC is sparse. Studies have shown that the increased expression of miR-483-5p in the plasma of patients with HCC suggests the occurrence of tumor as a potential biomarker HCC (Cong, 2016). Some studies have shown that the expression of miR-483-5p in HCC tissues is lower than that in surrounding liver tissues. However, the expression level and function of miR-483-5p in RHCC have not been reported.

Our study shows that the expression of miR-483-5p is significantly correlated with the recurrence interval of RHCC, and the results suggest that the increased expression of miR-483-5p can shorten the recurrence interval of HCC, which can be used as a predictor of short-term recurrence.

Cell function experiments show that miR-483-5p can promote the migration and invasion of HCC cells, but have no obvious effect on proliferation, and these results suggest that miR-483-5p can affect the metastasis and recurrence of HCC by promoting the migration and invasion abilities of HCC cells. *In vivo* experiments show that there is increased intrahepatic metastasis and microvascular invasion after miR-483-5p up-regulation, and vascular invasion and metastasis are not limited to the orthotopic liver lobe but are also present in the distant liver lobe, even in muscle tissue adjacent to the liver and lung. These results suggest that miR-483-5p overexpression can enhance the invasion and metastasis of HCC cells, and it leads to the short-term recurrence by the invasion-promoting effect.

MiR-483-5p is also involved in a variety of disease processes, such as facilitating infected lymphocytes through the blood-brain barrier, polycystic ovary syndrome, and liver fibrosis (Shen et al., 2013; Li et al., 2014; Chen et al., 2015; Shi et al., 2015; Curis et al., 2016), and it also plays an important role in the development of many tumors such as esophageal, oral squamous cancer, and lung adenocarcinoma (Song et al., 2014; Li et al., 2016; Xu et al., 2016).

The effect of miR-483-5p is achieved through effects on target genes. We predict several possible target genes according to bioinformatics analysis, and found the ALCAM gene, which is the direct target gene of miR-483-5p by luciferase reporter assay. Our experiments show that there are definite binding sites in miR-483-5p and the 3′ UTR region of ALCAM, and through the functional restoration experiment we finally confirm that miR-483-5p promotes the biologic function of tumor cell migration and invasion by regulating its target gene, ALCAM, in HCC.

ALCAM, as a member of the ‘immunoglobulin super family’, is known to be involved in cancer cell proliferation and migration. The expression and function of ALCAM vary in different tumors; in some tumors, it plays a role in promoting cancer, and in other tumors it plays a role in suppression; some research indicated it was highly expressed in gastric cancer and promoted the migration of tumor cells. The membranous ALCAM expression in gastric cancer tissue and serum was associated with shorter overall survival (Ye et al., 2015; Erturk et al., 2016). ALCAM can promote cell migration, invasion, and metastasis in endometrial carcinoma, and is the marker of recurrence in early-stage endometrioid endometrial cancer (Devis et al., 2016). However, the low expression of ALCAM indicates poor prognosis in infantile neuroblastoma (Wachowiak et al., 2016). ALCAM is also expressed in serum or other body fluids, and it can be used to predict the prognosis of tumors by detecting the changes in its expression. Studies have shown that serum ALCAM levels significantly increased in patients with breast cancer and can be used as a prognostic and predictive indicator (Al-Shehri and Abd El Azeem, 2015); the expression level of ALCAM in the urine of patients with bladder cancer can be used as a prognostic marker for survival (Egloff et al., 2016).

The positive staining of ALCAM was located at the cellular membrane or cytoplasm in immunohistochemistry stains, and 59.3% cases were positive on the cellular membrane, whereas 12.5% cases were positive in cytoplasm. The clinical significance and prognostic value of ALCAM positive located at membrane staining was more important than cytoplasm staining. ALCAM microarray analysis showed that the 1,3, and 5-year survival rates of ALCAM-positive cases were 75.8, 60.1, and 54.1%, respectively, and that of the ALCAM-negative cases were 80, 56.3, 50.2%, respectively; the median survival time was 37.8 months in the two groups, and the difference was not significant (*p* > 0.05). The 1,3, and 5-year recurrence rates of ALCAM-positive cases were 16.4, 31.3, and 50.1%, respectively, and the recurrence rates for the negative cases were 33.5, 56.1, and 67%, respectively. The median TTR of the positive group was 54.9 months, which was significantly higher than that of the negative group, which was 27.2 months (*p* < 0.01). Multivariate analysis showed that the risk of recurrence in ALCAM- negative patients was 1.876 times that of positive cases (*p* = 0.022, 95%confidence interval 1.096–3.211), which was an independent risk factor for postoperative recurrence. Factors associated with survival included HBeAb, albumin levels, venous invasion, and AFP, among which HBeAb, albumin levels, and venous invasion were independent risk factors for overall survival. And ALCAM is significantly associated with EMT in HCC cells, miR-483-5p-ALCAM can affect the invasion and metastasis of HCC by regulating the EMT process of HCC cells.

Our study describes the function and mechanism of miR-483-5p/ALCAM in RHCC. miR-483-5p promotes the migration and invasion of HCC cells, which leads to intrahepatic metastasis and distant metastasis and finally postoperative short-term recurrence; ALCAM is an independent risk factor of HCC recurrence and can be used as a biomarker for postoperative recurrence risk assessment of HCC.

## Statement of significance

miR-483-5p / ALCAM axis is an important regulator in invasion and metastasis and biomarker for recurrence risk assessment of HCC.

## Ethics statement

This study was carried out in accordance with the recommendations of “Committee on Ethics of Biomedicine, Second Military Medical University” with written informed consent from all subjects. All subjects gave written informed consent in accordance with the Declaration of Helsinki. The protocol was approved by the “Committee on Ethics of Biomedicine, Second Military Medical University.”

## Author contributions

W-MC, X-HH, X-YL, and ZC: designed the study; X-YL, ZC, X-YG, and DC: performed experiments; X-YL and W-MC: analyzed IHC data; JD and Y-JZ: helped to perform experiments; X-YL and W-MC: wrote the paper with comments from all authors.

### Conflict of interest statement

The authors declare that the research was conducted in the absence of any commercial or financial relationships that could be construed as a potential conflict of interest.

## Supplementary material

Figure S1 Effect of miR-483-5p and ALCAM on HCC cell proliferation. **(A)** miR-483-5p mimics had no obvious effects on cell proliferation of SK-Hep1. **(B)** miR-483-5p mimics had no obvious effects on cell proliferation of SMMC-7721. **(C)** Knockdown of ALCAM had no obvious effects on cell proliferation of SK-Hep1. **(D)** Knockdown of ALCAM had no obvious effects on cell proliferation of SMMC-7721.

Table S1 Primer sequence of candidate gene.

Table S2 siRNA sequences of ALCAM and NC.

Table S3 Univariate analysis of clinicopathological parameters associated with overall survival and disease-free survival.
