# Supplementary figures and images for: Predicting Value of ALCAM as a Target Gene of microRNA-483-5p in Patients with Early Recurrence in Hepatocellular Carcinoma
Source: Front Pharmacol. 2018 Jan 12;8:973. doi: 10.3389/fphar.2017.00973 (PMC5770356; doi:10.3389/fphar.2017.00973)

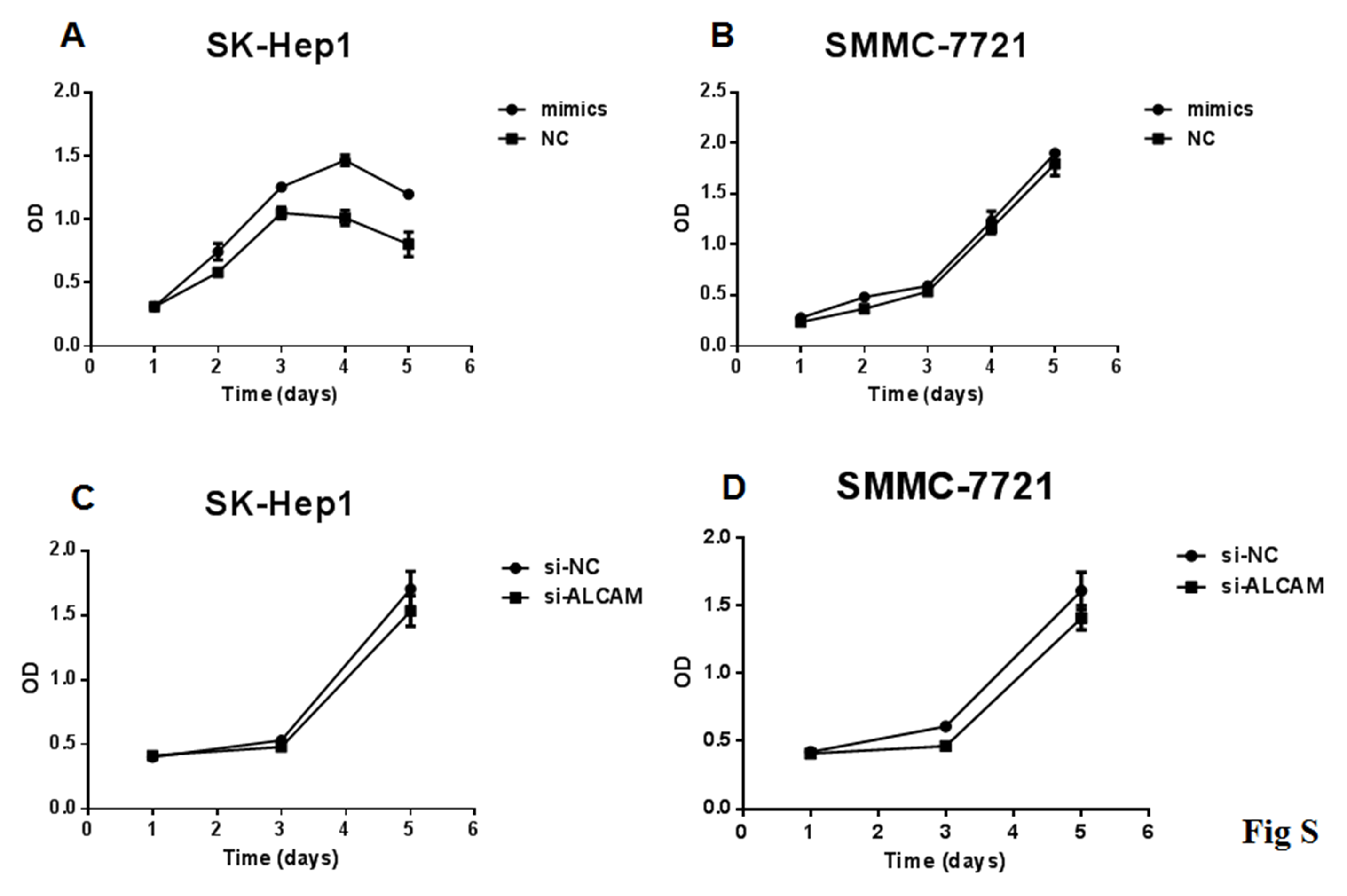

Supplement: Figure S1 — Effect of miR-483-5p and ALCAM on HCC cell proliferation. (A) miR-483-5p mimics had no obvious effects on cell proliferation of SK-Hep1. (B) miR-483-5p mimics had no obvious effects on cell proliferation of SMMC-7721. (C) Knockdown of ALCAM had no obvious effects on cell proliferation of SK-Hep1. (D) Knockdown of ALCAM had no obvious effects on cell proliferation of SMMC-7721. [file Image1.TIF]
