# Supplementary material for: Predicting Value of ALCAM as a Target Gene of microRNA-483-5p in Patients with Early Recurrence in Hepatocellular Carcinoma
Source: Front Pharmacol. 2018 Jan 12;8:973. doi: 10.3389/fphar.2017.00973 (PMC5770356; doi:10.3389/fphar.2017.00973)
Supplement: Table S1 — Primer sequence of candidate gene. [file Table1.DOCX]

**Table S1 Primer sequence of candidate gene**

| gene | Primer F | Primer R |
| --- | --- | --- |
| ABCF2 | AGAGATGCCAATGTCAGGGT | CATCCTGGCTTACAAGGAGC |
| ACBD6 | AGAGCTGTTTGGCCTTCATT | GCTCTACTTCACTGGGCCTG |
| ALCAM | AGCCATCACAGTTCACTATTT | TCTTTAGTCCTTCAACCTCC |
| ARHGDIA | CTGCTCAGCCATGCTCAAG | CGACGTTCGTCATTTAGTGC |
| ASPN | GGAGCACATACTCCTTCATGG | CATGGTGGATAACTTCTACTTTTAGGA |
| C1QL1 | CTGTTGCTGGCGTAGTCGTA | CCAGTATGTGGGCAGACCTC |
| CACNB1 | GGGACTTGATGAGCCTTTGA | TGACACCATCAATCACCCAG |
| CACYBP | CCAGCAACACCTTTACCTCTTC | TTTCCTGTTCCTCCTTCTGC |
| CD44 | CACGTGGAATACACCTGCAA | GACAAGTTTTGGTGGCACG |
| CDK5R2 | GCACCTCAGTCGATCCAAAT | CCACTTGTGTCAGCTTGCAT |
| CDKN2A | GTGAGAGTGGCGGGGTC | GTTACGGTCGGAGGCCG |
| CELSR2 | TCTCATCGGTGATGATGGTC | GACAACAACCGGCCTCTG |
| CXCL12 | TGGGCTCCTACTGTAAGGGTT | TTGACCCGAAGCTAAAGTGG |
| DLX1 | GCTTCTTGAACTTGGATCGC | CAGTACCTAGCTCTGCCGGA |
| ELK1 | GAAACTGCCACAGCGTCAC | CCGGAGCTGCCAACATT |
| ERBB3 | TCACACTCAGGCCATTCAGA | GTGCTGGGCTTGCTTTTC |
| HAND2 | GCACACGGGAGTGTCCTC | CCTTTGAGGCATCTGCTCC |
| KATNAL1 | CCTGCCATTTCTTTCCTCAG | TAGAGATCCTGCTGTTTGGC |
| MAPK3 | GGATGCCGATGACATTCTC | CATCAAGAAGATCAGCCCCT |
| MBTPS1 | TCAATCAACCACTGTGAGCC | AGTTGGGAGTAAACAGCCCC |
| MCAM | ACTTCCACCTCCACCAGCTC | GTCTGCGCCTTCTTGCTC |
| METAP2 | CCAAATCTCTTCACTTGCCTG | GGACAAGAATGCGAATACCC |
| MKNK1 | ATGGGAAGGGGTTCGCTACT | CAGGAGCTTTATTTCATTTGGG |
| MMP11 | TTCACAGGGTCAAACTTCCA | CCACTGACTGGAGAGGGGT |
| MPZ | TGTCGGTGTAAACCACGATG | CTATGGCTCCTGGGGCTC |
| NKX2-2 | GGAGCTTGAGTCCTGAGGG | TCTACGACAGCAGCGACAAC |
| NME4 | GCCTCTCAAAGCGCTGG | AGCCTGCTAGTGCGCCA |
| NUDT8 | GTGTGCACCACATCTTGGTC | GCTGTACACGCTGCGGTC |
| PDGFD | GGATGGTCTCATCTCTTCGG | CAGAGCGCATCCATCAAAG |
| PIP5K1A | AGAGGCATAAGGCACCTCAG | TCTTCGGTCGGTTTTTCATC |
| PIP5K1C | ACAGAACCTCTGTTGGGGC | CTGGAGGTACCGGACGAG |
| PJA2 | GCTTTCCTTACTAGCTGGTGGA | AGAAGAACGCTTAGCCCAGG |
| PLA2G5 | CTACAAGCCAGGAACCAAGC | GCAGACCCCTAGAGCAGGAT |
| PLAU | CCAGCTCACAATTCCAGTCA | TGACCCACAGTGGAAAACAG |
| PNMA3 | AGGGCGTGAGGCACATATC | ACCAGAAGGGACCTTGCC |
| POU4F2 | CTCTGGGAGACGATGTCCAC | ATGCGGAGAGCCTGTCTTC |
| PTMA | TTTCCTCATTAGCATTCCCG | AGACACCAGCTCCGAAATCA |
| PVRL1 | CAGTGCAGAACCACGTCTGT | GCTTGACCGCATTCTTCCT |
| RBM14 | CTTGCCCTTCACTTCTTTGC | ATCGAGTGTGACGTGGTGAA |
| RECK | TCACGGCACATTTGGTTATC | CTCCTTCTGCTGGCCGT |
| RHOA | AGCAAGCATGTCTTTCCACA | GAAGAGGCTGGACTCGGATT |
| RNF121 | CCTCCATTGCTCTTCTGGAG | AGTGGTGGAGGTGGAGGTT |
| SATB2 | CCACCTTCCCAGCTTGATT | TTAGCCAGCTGGTGGAGACT |
| SCRT1 | CCCACGTAGTCGCTGAGGTA | CAAACTTGACGCGTTCTCTTC |
| SELO | CTGGCGACTCTAGCTCCACT | CCAAGCTCCTGGAGACCAT |
| SLC12A5 | TCTGTGTCGGTGCTGTTGAT | GCCACCATGCTAAACAACCT |
| SLC29A4 | CCGTGTACCCGTAGAAGCTG | GTGGCTGCAGCTCTTCTCTC |
| SMAD4 | TTGATCCTTTGGAAACAGTGAA | GCCTTCCCACTCCCCTC |
| SMARCD1 | TTTTCTGATCATTCGGGTCA | TTTTCAGAGATCCCTCAGCG |
| SOX4 | AATGTATGTTTCCCCCTCCC | TCGCTGTCGGGTCTCTAGTT |
| SRF | ACACATGGCCTGTCTCACTG | CTTCAGCAAGAGGAAGACGG |
| TIMP2 | TCCTCTTGATAGGGTTGCCA | CGTTTTGCAATGCAGATGTA |
| TIMP3 | ATGGTGTAGACCAGCGTGC | AGGACGCCTTCTGCAACTC |
| TP53 | GCTCGACGCTAGGATCTGAC | GCTTTCCACGACGGTGAC |
| ZFYVE20 | CGATCATCCCCTTCTCGTT | AAGACCGTGATGTCAAAGGG |
