# Supplementary material for: Predicting Value of ALCAM as a Target Gene of microRNA-483-5p in Patients with Early Recurrence in Hepatocellular Carcinoma
Source: Front Pharmacol. 2018 Jan 12;8:973. doi: 10.3389/fphar.2017.00973 (PMC5770356; doi:10.3389/fphar.2017.00973)
Supplement: Table S2 — siRNA sequences of ALCAM and NC. [file Table2.DOCX]

**Table S2 siRNA sequences of ALCAM and NC**

| Small-interfering RNA | Sequences(5' to 3') |
| --- | --- |
| si-NC | UUCUCCGAACGUGUCACGU |
|  | GCATATGGAGATACCATTA |
| si-ALCAM | GCAATGCAACAGGAGACTA |
|  | CCTTGAATGTCTCTGCTAT |
