# Supplementary material for: Predicting Value of ALCAM as a Target Gene of microRNA-483-5p in Patients with Early Recurrence in Hepatocellular Carcinoma
Source: Front Pharmacol. 2018 Jan 12;8:973. doi: 10.3389/fphar.2017.00973 (PMC5770356; doi:10.3389/fphar.2017.00973)
Supplement: Table S3 — Univariate analysis of clinicopathological parameters associated with overall survival and disease-free survival. [file Table3.DOCX]

**Table S3 Univariate analysis of clinicopathological parameters associated with overall survival and disease-free survival**

| **Variables** | ***P* value: log–rank**  **Test for OS** | ***P* value: log–rank test for TTR** |
| --- | --- | --- |
| Gender  Male vs. Female | 0.801 | 0.452 |
| Age (year)  >50 vs.≤50 | 0.513 | 0.804 |
| Serum HbsAg  Pos vs. Neg | 0.900 | 0.387 |
| Serum HbsAb  Neg vs. Pos | 0.05* | 0.212 |
| Serum HbeAg  Pos vs. Neg | 0.123 | 0.036* |
| Serum HbeAb  Pos vs. Neg | 0.023* | 0.005** |
| HBV-DNA  Pos vs. Neg | 0.784 | 0.841 |
| Serum AFP (ng/ml)  >20 vs.≤20 | 0.030* | 0.105 |
| Serum TB (μmol/L)  >17.1 vs.≤17.1 | 0.687 | 0.840 |
| Serum albumin (g/L)  <40 vs. 40-55 | 0.008** | 0.222 |
| ALT (U/L)  >40 vs. 5-40 | 0.164 | 0.844 |
| AST (U/L)  >40 vs. 8-40 | 0.000** | 0.355 |
| Portal hypertension  Yes vs. No | 0.390 | 0.905 |
| Ascites  Yes vs. No | 0.590 | 0.815 |
| Vascular invasion  Yes vs. No | 0.001** | 0.173 |
| ALCAM  Neg vs. Pos | 0.594 | 0.01** |
| Cirrhosis  Yes vs. No | 0.220 | 0.576 |
| Tumor size  >3cm vs. ≤3cm | 0.183 | 0.567 |
| Capsule  No vs. Yes | 0.098 | 0.536 |
| E–S grade  Ⅲ-Ⅳ vs. I-Ⅱ | 0.348 | 0.985 |

OS: Overall survival; TTR: Time to recurrence (**P* < 0.05, ***P* < 0.01)
